# Effect of different stripping techniques on pulpal temperature: in vitro study

**DOI:** 10.1590/2177-6709.24.1.039-043.oar

**Published:** 2019

**Authors:** Megha Sehgal, Payal Sharma, Achint Juneja, Piush Kumar, Anubha Verma, Vikas Chauhan

**Affiliations:** 1 I.T.S. Dental College, Department of Orthodontics and Dentofacial Orthopedics (Muradnagar, India).

**Keywords:** Proximal stripping, Frictional heat, Pulpal temperature

## Abstract

**Introduction:**

Proximal stripping of enamel is a routine clinical procedure employed in orthodontics to create space or for balancing tooth size discrepancies. This procedure may result in heat transfer to the pulp, predisposing it to histopathological changes and necrosis of the pulp tissue.

**Objective:**

To measure the temperature changes in the pulp chamber during different stripping procedures.

**Methods:**

80 proximal surfaces of 40 extracted human premolar teeth were stripped using four techniques: diamond burs in air-rotor handpiece with air-water spray; diamond burs in micromotor handpiece, with and without a coolant spray; and hand-held diamond strips. A J-type thermocouple connected to a digital thermometer was inserted into the pulp chamber for evaluation of temperature during the stripping procedure.

**Results:**

An increase in the pulpal temperature was observed for all stripping method. Diamond burs in micromotor handpiece without coolant resulted in the higher increase in temperature (3.5^o^C), followed by hand-held diamond strips (2.8^o^C), diamond burs in air-rotor with air-water spray (1.9^o^C); and the smallest increase was seen with diamond burs in micromotor handpiece with coolant (1.65^o^C). None of the techniques resulted in temperature increase above the critical level of 5.5^o^C.

**Conclusion:**

Frictional heat produced with different stripping techniques results in increase in the pulpal temperature, therefore, caution is advised during this procedure. A coolant spray can limit the increase in temperature of the pulp.

## RESUMO

**Introdução:**

o desgaste proximal do esmalte é um procedimento clínico rotineiro utilizado na Ortodontia para se criar espaços ou equilibrar discrepâncias de tamanho dentário. Esse procedimento pode resultar em transferência de calor para a polpa, predispondo-a a mudanças histopatológicas e necrose do tecido pulpar.

**Objetivo:**

medir as mudanças de temperatura na câmara pulpar durante diferentes procedimentos de desgaste interproximal.

**Métodos:**

80 superfícies proximais de 40 pré-molares humanos foram desgastadas utilizando-se quatro técnicas diferentes: brocas diamantadas em motor a ar (alta rotação) com *spray* de água e ar; brocas diamantadas em micromotor (baixa rotação) com e sem *spray* de resfriamento; e tiras diamantadas manuais. Um par termoelétrico do tipo J conectado a um termômetro digital foi inserido na câmara pulpar para avaliação da temperatura durante o desgaste proximal.

**Resultados:**

foi observado um aumento da temperatura da câmara pulpar em todos os métodos de desgaste proximal. As brocas diamantadas em micromotor sem resfriamento foram responsáveis pelo maior aumento da temperatura (3,5^o^C), seguidas pelas lixas diamantadas manuais (2,8^o^C) e brocas diamantadas em motor a ar (alta rotação) com *spray* de água e ar (1,9^o^C). O menor aumento foi observado com as brocas diamantadas em micromotor (baixa rotação) com resfriamento (1,65^o^C). Nenhuma das técnicas elevou a temperatura acima do nível crítico de 5,5^o^C.

**Conclusão:**

o aquecimento friccional produzido pelas diferentes técnicas de desgaste proximal levou ao aumento da temperatura da câmara pulpar; assim, cuidados devem ser tomados durante esse procedimento. O *spray* de água e ar pode limitar o aumento da temperatura da polpa.

## INTRODUCTION

Proximal stripping involving enamel reduction is a routine clinical procedure in orthodontics used to gain space for correction of mild crowding, rotation or tooth recontouring. According to Sheridan and Ledoux,^1^ 6.4 mm of space can be gained from proximal stripping of upper molars and premolars.

The creation of enamel irregularities subsequent to proximal stripping may increase the susceptibility of these teeth to the accumulation of plaque, consequently with higher chances of caries and periodontal disease. However, this has been contested by some authors who concluded that stripped teeth were not more susceptible to caries or periodontal damage.^1^ ^,^ ^2^

Another side effect of this procedure is the possibility of increase in pulpal temperature following stripping, which can lead to histopathological changes and pulpal necrosis. A classic study by Zach and Cohen^3^ performed in teeth of primates showed that an increase of 5.5°C in the pulp chamber can cause considerable damage, compromising the pulp health and producing irreversible inflammation in 40% of the specimens tested. Therefore, Zachrisson et al^4^ and Sheridan^5^ emphasized the importance of cooling during stripping. Sheridan^5^ suggested using water sprays to prevent the possible damaging effect of frictional heat during air rotor stripping (ARS). However, the use of cooling water spray hampers visibility during the stripping procedure, which is important to prevent enamel and periodontal damage.

Currently there are three major techniques for IER (Interproximal Enamel Reduction): Air-rotor stripping technique with ﬁne tungsten-carbide or diamond burs and diamond-coated strips; handpiece or contra-angle-mounted diamond-coated disks; and hand-held or motor-driven abrasive metal strips. The type of procedure employed may affect the heat produced during stripping.

Therefore, the aim of this *in vitro* study was to measure temperature changes in the pulp chamber during different stripping procedures. 

## MATERIAL AND METHODS

This study was approved by the Institutional Ethical Committee of the ITS Centre for Dental Studies & Research (Director - PG Studies/ITSCDSR/L/2017/17). A sample size calculation was done for the level of significance a = 5% and power = 80%. By surveying the literature, the expected mean temperature difference and pooled S.D. between two groups was found to be 0.748 and 1.189, respectively, which resulted in a required sample size of 19 for each group. 

Forty (*n*= 40) intact premolars, extracted for orthodontic purposes, were stored in normal saline until being fixed by the root portion to a colorless self-curing acrylic resin support. The dental crown remained fully exposed, for implementing the stripping procedures. The teeth were randomly divided into four groups of 10 teeth each, based on the method used for stripping:

» Group 1: stripping with diamond burs (TC - 12, S.S. White, USA) in an air-rotor handpiece (NSK, Japan) with air-water spray.
» Group 2: stripping with diamond burs (TC - 12, S.S. White, USA) in micromotor handpiece (NSK, Japan) without coolant.
» Group 3: stripping with diamond burs (TC - 12, S.S. White, USA) in micromotor handpiece (NSK, Japan) with water coolant.
» Group 4: stripping with hand-held diamond strips (Steel Abrasive Strips, Produits Dentaires S.A., Switzerland).

A wide cavity of 2.00 mm in diameter was made with spherical diamond burs on the occlusal surface of premolars, following the pulp chamber. Pulp tissue debris were removed with a manual curette and the pulp chamber, irrigated with 1% sodium hypochlorite. Thereafter, the pulp chamber was slightly dried and filled with a silicon heat transfer (Asian Test Equipments, Uttar Pradesh, India), to facilitate heat transfer.

**Figure 1:**
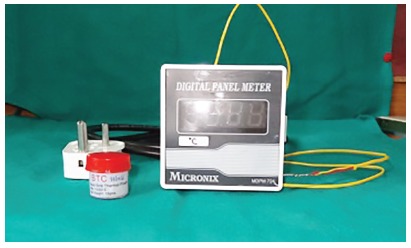
J-type thermocouple connected to digital thermometer and silicon heat transfer material.

**Figure 2:**
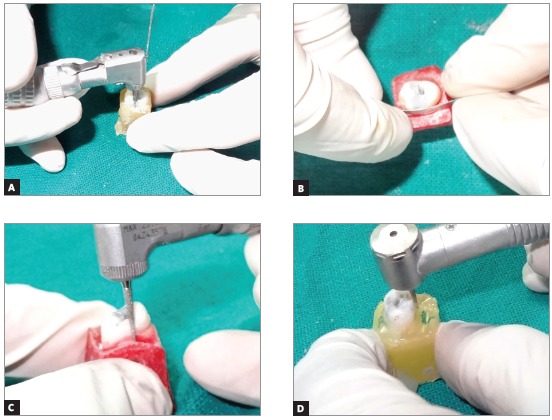
Proximal stripping with different techniques: A) Stripping with diamond burs in micromotor handpiece with water as coolant; B) Stripping with hand-held stripper; C) Stripping with diamond burs in air-rotor; D) Stripping with diamond burs in micromotor handpiece without coolant.

**Figure 3:**
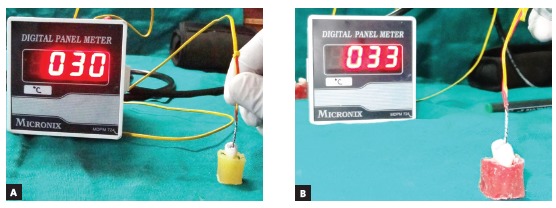
Temperature measured on digital thermometer connected to J-type thermocouple: A) before stripping ; B) after stripping.

A J-type thermocouple (Asian Test Equipments, Uttar Pradesh, India) with 1.60-mm diameter was inserted into the pulp chamber and connected to a digital thermometer to measure the temperature, both before and after the stripping procedure (Fig 1). Teeth were randomly divided by sides, in order to perform the 0.5-mm stripping of interproximal enamel on the same tooth using two different techniques (Fig 2). Thus, the sample of 40 teeth provided 80 readings, 20 for each group. After measuring the initial temperature, the stripping procedure was performed by a single examiner, in order to avoid intra-examiner variation, according to the method assigned. Both proximal surfaces were stripped 0.5 mm each, as measured using vernier calipers. The temperature was recorded again after the stripping procedure. Temperatures produced before and after stripping were recorded on the thermometer display (Fig 3). Only one proximal surface was stripped at a time, and the tooth was allowed to reach room temperature before the contralateral surface was stripped. The thermometer display was covered during the procedure and the temperature revealed only after all the stripping had been performed.

After the stripping of every five teeth, diamond burs were replaced, and the metal hand-held strippers were changed after the surface was worn. 

The data obtained from all measurements were processed with SPSS 16 software. Statistics of all the four experimental groups are shown in Table 1. Analysis of variance test (ANOVA) followed by Tukey’s HSD *post-hoc* test was used to detect if there was any difference in the temperature increase produced with the different stripping procedures.

**Table 1:** Statistics analysis of temperature changes in the experimental groups.

Groups	n	Temperature before stripping (^o^C) Mean ± S.D.	Temperature after stripping (^o^C) Mean ± S.D.	Temperature difference (^o^C) Mean ± S.D.	Standard error (temperature difference)	95% Confidence Interval for mean (temperature difference)		Sig. (Between groups)
						Lower bound	Upper bound	
Group 1^a^	20	30.40 ± 1.23	32.30 ± 1.72	1.90 ± 1.07	1.08	1.40	2.40	0.00
Group 2^b^	20	29.95 ± 1.145	33.45 ± 1.32	3.50 ± 0.95	0.95	3.06	3.94	
Group 3^a^	20	29.75 ± 0.85	31.40 ± 1.05	1.65 ± 0.81	0.81	1.27	2.03	
Group 4^b^	20	29.90 ± 1.16	32.70 ± 1.22	2.80 ± 0.41	0.41	2.60	2.99	

* Different letters denote statistically significant difference between groups.

## RESULTS

The highest increase in temperature of the pulp was seen for stripping with diamond burs in a micromotor handpiece without coolant (3.5˚C), followed by hand stripping with diamond strips, air-rotor stripping with diamond burs and water spray and, finally, by stripping with diamond burs in micromotor handpiece with coolant. 

## DISCUSSION

The frictional heat generated by various stripping techniques may present a risk factor for the dental pulp. This*in vitro* study aimed at evaluating the temperature changes produced by various stripping techniques, namely: air-rotor stripping with diamond burs and water spray, diamond burs in micromotor handpiece with and without coolant, and hand-held diamond strips. 

A thermocouple unit was used to evaluate changes in pulpal temperature because of its reliability and accuracy, as reported in previous studies.^6^ ^-^ ^10^

It was found that 50% of proximal enamel is the maximum amount that can be stripped without causing dental and periodontal problems.^11^ According to Stroud et al,^12^ 9.8 mm of additional space may be provided by enamel reduction of mandibular premolars and molars. Following the latest updates of guidelines for contemporary air-rotor stripping, an amount of 1 mm (0.5 mm per proximal surface) can be removed from the contact points of the buccal section.^13^ In the present study, the amount of stripping was hence standardized to be 0.5 mm, which was controlled by measuring tooth widths before and after stripping, with a digital vernier caliper, similar to the study performed by Pereira et al.^14^ Different method was used by Baysal et al,^6^ who counted the number of strokes (20 in case of hand-held strips) or timed the procedure (10 seconds) when stripping with perforated disks and tungsten carbide burs.

The results of the present study revealed that the temperature of the pulp varied with the type of stripping procedure used, and the difference was statistically significant. Stripping performed with diamond burs in a micromotor handpiece without the use of a coolant produced the maximum increase in temperature, with an average of 3.5˚C; followed by stripping with hand-held diamond strips, with a mean increase of 2.8˚C. Diamond burs in an air-rotor (1.9˚C) or micromotor handpiece (1.65˚C) produced less heating when used with a cooling water spray. 

A similar study by Baysal et al^6^ also showed a significant increase in pulp temperature in different groups of teeth (incisors, canines and premolars) when stripped with perforated stripping discs, hand-held strippers and tungsten carbide burs without any coolant. Results of this study are in accordance with those of Pereira et al,^14^ who also demonstrated an increase in pulpal temperature in different tooth specimens (incisors, premolars and molars) with perforated diamond discs and hand-held strippers. In both these studies, perforated discs produced higher increase in the temperature than metal hand-held strippers. The metal strips were therefore recommended by the authors as the safest choice. In the present study, the diamond hand-held strips produced more heat than the methods using water spray.

The increase in temperature varied from 0 to 5˚C, with an average change of 2.46˚C. Although an increase in pulpal temperature was seen in all the groups, the critical threshold of 5.5˚C suggested by Zach and Cohen^3^ was not reached. In the study by Baysal et al,^6^ the critical temperature was reached when the mandibular incisors were stripped using tungsten carbide burs with a high speed handpiece, with an increase of 5.63 ± 1.63˚C. This may have been caused by the thin enamel on incisors. The same method on premolars resulted in an increase of 3.65 ± 1.67˚C in temperature. This is similar to the average increase of 3.5˚C obtained in the present study when stripping with diamond burs in an air-rotor handpiece without coolant spray in premolars. However, the maximum increase produced with this method was 5˚C, which is quite close to the threshold value. In the study by Pereira et al,^14^ the highest temperature was produced by perforated stripping discs in molars (3.2˚C) followed by 3.1˚C in premolars and incisors.

Some authors^4^ ^,^ ^5^ have suggested that cooling techniques, such as an air-water spray, were effective in limiting the temperature increase in the pulp chamber. However, according to some of these authors^4^ the use of water for cooling hampers visibility during the procedure. The results of the present study support the use of water spray as a safety measure, since the increase in temperature may be greater in young permanent teeth, compared to adult teeth tested in *in vitro* studies. The cooling with water limited the generation of heat even with the use of high speed air-rotor handpiece.

The *in vitro* nature of this study did not allow evaluation of factors such as pulpal vascularity, presence of pain during the stripping procedure leading to an increase in the blood flow and heat conduction within the tooth due to the effect of blood circulation in the pulp chamber and fluid motion in the dentinal tubules. Moreover, only the premolar teeth were evaluated. Previous studies^6^ ^,^ ^14^ included different groups of teeth such as incisors and molars, to account for the variable thickness of enamel. No difference in temperature variation was found when Pereira et al.^14^ compared different groups of teeth. The authors suggested that the higher bucco-lingual volume of enamel found in molars required a greater amount of time for the removal of the same thickness of enamel. Pulpal response may also vary in carious or restored teeth, hypocalcified or fluorosed enamel and reversibly inflamed pulpal tissue. The above stated factors could also not be evaluated because only intact teeth were included in the study.

## CONCLUSION

All stripping methods resulted in an increase in the pulpal temperature, however none of the procedures showed an increase above the critical level of 5.5 ˚C.
Diamond burs in a micromotor handpiece without the use of coolant resulted in the higher increase in pulpal temperature.
It is recommended to use a cooling air-water spray to limit the generation of heat during stripping procedures.
